# Urea-Triazone N Characteristics and Uses

**DOI:** 10.1100/tsw.2001.356

**Published:** 2001-11-21

**Authors:** John G. Clapp

**Affiliations:** Technical Agricultural Products, Research and Development, Tessenderlo Kerley, Inc, Greensboro, NC 27405, USA

**Keywords:** nitrogen fertilizer, urea-triazone, ammonia volatilization, nitrogen leaching, foliar nitrogen, turf

## Abstract

Urea-triazone nitrogen (N) is a stable solution resulting from a controlled reaction in aqueous medium of urea, formaldehyde, and ammonia which contains at least 25% total N. This N source contains no more than 40%, nor less than 5%, of total N from unreacted urea and not less that 40% from triazone. All other N shall be derived from water-soluble dissolved reaction products of the above reactants. It is a source of slowly available N. The rate of mineralization of urea-triazone is about 66% that of urea after 8 days when incorporated in a Munjor sandy loam. Ammonia volatilization losses of N applied as urea-triazone were about 41% of those from urea on a Cecil sandy loam in the first week after application. N leaching losses through saturated Yolo loam columns of urea-triazone were about two thirds that of urea or nitrate N. This N source has proven to be a safer and more effective material for direct application on plant foliage. Tomato growth was enhanced with foliar application of urea-triazone relative to that obtained from ammonium nitrate or urea. The stability of this N source from potential losses via ammonia volatilization and nitrate leaching when soil applied is also documented by results from university trials.

## INTRODUCTION

Urea-triazone liquid nitrogen (N) fertilizers were developed by the Triazone Corporation in the mid 1980s. Triazone and its analogues are produced by reacting urea or substituted urea, an aldehyde, and ammonia or primary amine under controlled conditions whereby all the aldehyde and ammonia or primary amine reacts. Some unreacted urea remains in the solution. The current commercial urea-triazone N fertilizer product, N-SURE, is produced by reacting urea, formaldehyde, and ammonia. Table 1 shows the characteristics of this product.

Urea-triazone solutions contain heterocyclic, organic N compounds. The predominate species are tetrahydro-s-triazone and 5-methyleneuriedo-2-oxohexahydro-s-triazine (Fig. 1).

Urea-triazone N solution is defined by the Association of American Plant Food Control Officials (AAPFCO) as a stable solution resulting from controlled reaction in aqueous medium of urea, formaldehyde, and ammonia which contains at least 25% total N[1]. The solution shall contain no more than 40%, nor less than 5%, of total N from unreacted urea and not less than 40% from triazone. All other N shall be derived from water-soluble dissolved reaction products of the above reactants. It is a source of slowly available N.

Urea-triazone N solutions have unique properties that are desirable for direct application to plant foliage. Products containing triazone N remain on the plant tissue surface in a liquid phase much longer than conventional-based urea N products. When foliar N is applied with all urea-based products, the solution water evaporates very quickly, often within 30 min of application, leaving a crystalline residue on the tissue surface. Foliar N absorption under these conditions is limited to a few minutes after application.

## LABORATORY EVALUATION

In order to determine the N-release pattern from urea-triazone solutions, a study was initiated at Kansas State University to determine the mineralization to ammonium and nitrate N. Several urea-triazone formulations were evaluated in comparison to a 100% urea solution. The materials were thoroughly mixed with a Munjor sandy loam at 100 mg N $kg^{-1}$ of soil. This soil had a pH of 8.0 and a water content of 0.202 kg $kg^{-1}$ of dry soil. The N-release pattern was evaluated at three incubation temperatures, 12, 22, and 32ºC.

tetrahydro-s-triazone 5-methyleneuriedo-2-oxohexahydro-s-triazine

**FIGURE 1.** Predominate N compounds in urea-triazone solutions.

**TABLE 1**
**N-SURE Technical Information**

| Characteristics | % |
|---|---|
| Guaranteed total N concentration | 28 |
| Distribution of N forms (% of total) | |
| Slow-release N | 72 |
| Urea N | 28 |
| Mean properties | |
| Specific gravity | 154 g $l^{-1}$ |
| pH | 9.5 |
| Salting-out temperature | <–18°C |

*Note:* Tessenderlo Kerley, Inc., Phoenix, Arizona.

The N-release pattern for a urea-triazone formulation, which later became the commercial product (N-SURE), is compared to the 100% urea treatment in Table 2. These data illustrate the N-release pattern at 22ºC as measured by the formation of ammonium and nitrate N for 24 days after soil incorporation.

The rate of mineralization for the urea-triazone solution (75% reacted N and 25% unreacted urea) was considerably slower than for the 100% urea solution. All of the N in the 100% urea treatment was converted to ammonium and nitrate after 2 days, while only 48% of the N in the urea-triazone formulation had been converted. Results from this study support the characterization of N-SURE as a source of slowly available N.

Kissel and Cabrera evaluated urea-triazone solutions for ammonia volatilization under laboratory conditions[2]. The loss

**TABLE 2**
**Mineralization of Urea and Urea-Triazone**

| Material | Days After Application | % of Applied N as Ammonium | % of Applied N as Nitrate | Total |
|---|---|---|---|---|
| 100% Urea | 2 | 84 | 17 | 101 |
| Urea-triazone | 2 | 32 | 16 | 48 |
| 100% Urea | 4 | 50 | 38 | 88 |
| Urea-triazone | 4 | 20 | 35 | 55 |
| 100% Urea | 8 | 5 | 84 | 89 |
| Urea-triazone | 8 | 2 | 57 | 59 |
| 100% Urea | 24 | 0 | 88 | 88 |
| Urea-triazone | 24 | 1 | 63 | 64 |

*Note:* Release when incubated at 22ºC. Previously unpublished data supplied by Dr. D.E. Kissel, Department of Soil Science, University of Georgia.

of N as ammonium volatilization from a surface application of urea fertilizers has been well documented by many[3,4,5,6,7]. These losses can be substantial. Kissel and Cabrera found that a urea-triazone solution containing 62% of the N in reacted form lost 7.5% of the applied N from ammonia volatilization, as compared to 16.7% from the 100% urea solution[2]. Since the urea-triazone solution contained unreacted urea (38%), the loss as ammonia volatilization would come from urea rather than the reacted N species in the formulation.

A second laboratory trial was conducted at the University of Georgia to evaluate urea-triazone solutions for losses via ammonia volatilization. In this study, N was applied to the surface of a Cecil sandy loam soil at a rate of 5 g N $m^{-2}$, with the temperature maintained at 25°C. Volatilization losses from a urea-triazone formulation containing 75% reacted N and 25% unreacted urea was compared with 100% urea for 9 days (Table 3).

In this trial, the loss from the 100% urea solution was substantial, with over 50% of the N being lost at 3 days after application. During this same period, the urea-triazone solution lost only 13% of the N from ammonia volatilization.

The movement of urea-triazone N through soil was performed using a 17-cm length column of Yolo loam, collected from the upper 2 cm under a rye grass turf, and screened to a particle size of between 1 and 2 mm. The column was exhaustively leached before N treatment in order to remove residual N. The N sources were added in solution to the top of the column and leaching was initiated using a head of water. N was added at a rate of 5 g N $m^{-2}$, which would be a typical rate applied to turf grasses. N sources included a nitrate, urea, and N-SURE, a commercial urea-triazone solution containing 28% total N.

The soil in these columns was very well structured, and permitted a rapid saturated conductivity of about 1 soil pore volume per 5 min. Leachate was then collected from samples representing one, two, three, and four times the soil pore volume. These samples were then analyzed for leached N. Urea-triazone N-SURE is less likely to leach through the soil profile (Table 4).

**TABLE 3**
**N Volatilized from Urea and Urea-Triazone**

| Days After Application | % of Applied N Volatilized: Urea | % of Applied N Volatilized: Urea-triazone |
|---|---|---|
| 1 | 10 | 5 |
| 2 | 41 | 10 |
| 3 | 57 | 13 |
| 4 | 64 | 16 |
| 5 | 68 | 21 |
| 6 | 69 | 27 |
| 7 | 70 | 29 |
| 8 | 71 | 31 |
| 9 | 71 | 33 |

*Note:* Temperature maintained at 25°C. Previously unpublished data supplied by Drs. W.L. Hargrove, R.N. Carrow, and J. Hall, Department of Soil Sciences, University of Georgia.

**TABLE 4**
**Nitrogen Leaching Losses**

| | N Leached by Pore Volume (%) | | | |
|---|---|---|---|---|
| **N Source** | **1** | **2** | **3** | **4** |
| Nitrate | 68 | 98 | 100 | 100 |
| Urea | 43 | 83 | 90 | 92 |
| N-SURE | 25 | 50 | 60 | 63 |

*Note:* Applied at rate of 5 g N $m^{-2}$. N-SURE (28-0-0) contained 72% reacted N and 28% unreacted urea. Previously unpublished data supplied by Dr. D.C. Bowman, Department of Crop Science, North Carolina State University.

**TABLE 5**
**Tomato Growth Response to Foliar N Sources**

| N Source | Fresh Weight (g) | Dry Weight (g) | Height (cm) |
|---|---|---|---|
| Ammonium nitrate | 7.16 | 1.36 | 23.6 |
| Urea | 6.68 | 1.25 | 22.3 |
| N-SURE | 8.17 | 1.60 | 24.5 |
| None | 5.94 | 1.19 | 18.7 |

*Note:* Previously unpublished data supplied by Dr. D.W. Reed, Department of Horticultural Sciences, Texas A&M University.

Nitrate N moved as expected through the soil profile in greater quantities than urea or N-SURE. As noted, at the 1-pore volume rate, leachate from the nitrate N source contained 2.7 times as much N as from N-SURE. At the 4-pore volume rate, the amount of N leached from N-SURE was less than the 1-pore volume rate for the nitrate source and less than that from the 2-pore volume rate for the urea source.

These results illustrate that a urea-triazone solution is less likely to leach through the soil profile than nitrate or urea when applied at rates equivalent to average N rates for turf. However, the soil used in this study was bare soil. Under an active turf environment, less N from all sources would leach through the soil profile.

Urea-triazone solutions have been evaluated under laboratory conditions as a foliar N source. N sources included N-SURE (the commercial urea-triazone product), ammonium nitrate, and urea. Tomato plants were treated at 500 ppm N until runoff for weeks 1 and 2, at 1000 ppm N at week 3, and 2000 ppm N at week 4. In this trial, a cover was placed under the plants to prevent any of the spray or runoff from coming in contact with the soil. The tomato plant height was measured at week 5, followed by harvesting the entire plants.

Results from this trial show that plant growth was stimulated more from N-SURE than from ammonium nitrate or urea (Table 5). When compared to the control, tomato dry weight increased by 5, 14.3, and 34.5% for urea, ammonium nitrate, and N-SURE, respectively. The minimal growth response for urea may have been related to N losses via ammonium volatilization.

## FIELD EVALUATION

The first field evaluation of a urea-triazone N solution was on St. Augustine and Bermuda grass turf in Florida[8]. This formulation contained 42% of the total N solution as triazone and was compared with a 100% urea solution and a urea-ammonium nitrate (UAN) solution at rates of 0.73, 1.46, and 2.93 kg N/100 M2. Leaf injury ratings showed that urea-triazone was much safer to these turf grasses than either a 100% urea solution or UAN. Other field trials also document that urea-triazone (N-SURE) is a desirable N source for application to turf grasses[9,10,11].

Urea-triazone N solutions were evaluated for potential leaf injury on agronomic and horticultural crops at 61 commercial grower sites throughout the U.S. Foliar spray solutions containing urea-triazone were used at concentrations ranging from 1.5 to 15.7 % N. Safe N concentrations for urea-triazone N products ranged from 1.5% for crops such as sweet corn, apple, cherry, and pear, and up to 15.7% for nursery root stocks. These rates were much safer on crop foliage than ammonium, nitrate, and/or all urea-based foliar N fertilizer products than reported in the literature[12].

## SUMMARY

Urea-triazone N solutions have been evaluated by a number of researchers over the last 15 years. This form of N has proven to be a source of fertilizer N with slow release characteristics, more stable than a 100% urea solution when applied to plant or soil surfaces and less likely to leach through the soil profile. It is safer to plant foliage than urea or urea-ammonium nitrate solutions. The commercial urea-triazone product, N-SURE, containing 28% total N with 72% of the N in a reacted form from controlled reactions of urea, formaldehyde, and ammonia, is an efficient N source for application to agronomic crops and especially to turf grasses.

| | | |
|---|---|---|
| Received: | July | 23, 2001 |
| Revised: | October | 4, 2001 |
| Accepted: | November | 2, 2001 |
| Published: | November | 21, 2001 |
